# Supplementary material for: Who tweets climate change papers? investigating publics of research through users’ descriptions
Source: PLoS One. 2022 Jun 3;17(6):e0268999. doi: 10.1371/journal.pone.0268999 (PMC9165795; doi:10.1371/journal.pone.0268999)
Supplement: S1 File — (ZIP) [file pone.0268999.s001.zip › S1 Table.pdf]

Title	Publication Year	Total number of users	Academic coverage		Academic overlaps					
			N of Academic assign.	% of Academic assign.	% No overlap	% Communication overlap	% Political overlap	% Professional overlap	% Personal overlap	% Org|Pub|Bot overlap
Total		19783	5545	28.0	32.2	13.1	11.8	15.7	29.4	28.3
Climate change in the Fertile Crescent and implications of the recent Syrian drought	2015	1760	245	13.9	22.4	22.4	20.8	18.0	40.8	22.0
The geographical distribution of fossil fuels unused when limiting global warming to 2 degrees C	2015	1265	292	23.1	26.4	13.7	24.0	20.2	33.6	24.3
Accelerating extinction risk from climate change	2015	749	152	20.3	26.3	17.1	19.7	17.8	38.2	23.7
Health and climate change: policy responses to protect public health	2015	481	126	26.2	19.0	13.5	18.3	27.8	36.5	32.5
Climate change impacts on bumblebees converge across continents	2015	337	91	27.0	30.8	13.2	6.6	17.6	30.8	34.1
Analysis and valuation of the health and climate change cobenefits of dietary change	2016	659	166	25.2	24.7	15.7	15.1	20.5	41.6	27.7
Oxygen isotope in archaeological bioapatites from India: Implications to climate change and decline of Bronze Age Harappan civilization	2016	537	56	10.4	28.6	25.0	16.1	16.1	25.0	19.6
Global and regional health effects of future food production under climate change: a modelling study	2016	347	75	21.6	30.7	14.7	18.7	21.3	30.7	29.3
Ecological networks are more sensitive to plant than to animal extinction under climate change	2016	276	132	47.8	36.4	10.6	6.8	8.3	26.5	31.1
Assessing the Performance of EU Nature Legislation in Protecting Target Bird Species in an Era of Climate Change	2016	238	62	26.1	30.6	12.9	16.1	16.1	33.9	17.7
