# Supplementary material for: Who tweets climate change papers? investigating publics of research through users’ descriptions
Source: PLoS One. 2022 Jun 3;17(6):e0268999. doi: 10.1371/journal.pone.0268999 (PMC9165795; doi:10.1371/journal.pone.0268999)
Supplement: S1 File — (ZIP) [file pone.0268999.s001.zip › S3 Table.pdf]

Title	Publication Year	Total number of users	Political coverage		Political overlaps					
			N of Political assign.	% of Political assign.	% No overlap	% Academic overlap	% Communication overlap	% Professional overlap	% Personal overlap	% Org|Pub|Bot overlap
Total		19783	2564	13.0	28.0	25.6	12.4	21.6	33.2	19.4
Climate change in the Fertile Crescent and implications of the recent Syrian drought	2015	1760	337	19.1	32.0	15.1	17.2	18.1	46.6	12.5
The geographical distribution of fossil fuels unused when limiting global warming to 2 degrees C	2015	1265	301	23.8	29.9	23.3	11.6	18.3	30.2	16.6
Accelerating extinction risk from climate change	2015	749	138	18.4	37.0	21.7	15.9	14.5	44.9	10.1
Health and climate change: policy responses to protect public health	2015	481	95	19.8	26.3	24.2	11.6	21.1	38.9	32.6
Climate change impacts on bumblebees converge across continents	2015	337	37	11.0	32.4	16.2	2.7	16.2	35.1	24.3
Analysis and valuation of the health and climate change cobenefits of dietary change	2016	659	133	20.2	38.3	18.8	15.0	20.3	32.3	12.8
Oxygen isotope in archaeological bioapatites from India: Implications to climate change and decline of Bronze Age Harappan civilization	2016	537	44	8.2	31.8	20.5	15.9	27.3	31.8	9.1
Global and regional health effects of future food production under climate change: a modelling study	2016	347	56	16.1	28.6	25.0	8.9	28.6	41.1	23.2
Ecological networks are more sensitive to plant than to animal extinction under climate change	2016	276	25	9.1	28.0	36.0	4.0	16.0	24.0	20.0
Assessing the Performance of EU Nature Legislation in Protecting Target Bird Species in an Era of Climate Change	2016	238	31	13.0	25.8	32.3	9.7	29.0	29.0	12.9
