# Supplementary material for: Who tweets climate change papers? investigating publics of research through users’ descriptions
Source: PLoS One. 2022 Jun 3;17(6):e0268999. doi: 10.1371/journal.pone.0268999 (PMC9165795; doi:10.1371/journal.pone.0268999)
Supplement: S1 File — (ZIP) [file pone.0268999.s001.zip › S5 Table.pdf]

Title	Publication Year	Total number of users	Personal coverage		Personal overlaps					
			N of Personal assign.	% of Personal assign.	% No overlap	% Academic overlap	% Communication overlap	% Political overlap	% Personal overlap	% Org|Pub|Bot overlap
Total		19783	4939	25.0	32.0	33.0	15.6	17.3	20.6	15.1
Climate change in the Fertile Crescent and implications of the recent Syrian drought	2015	1760	611	34.7	42.9	16.4	17.8	25.7	17.3	10.3
The geographical distribution of fossil fuels unused when limiting global warming to 2 degrees C	2015	1265	350	27.7	24.9	28.0	17.4	26.0	20.9	16.0
Accelerating extinction risk from climate change	2015	749	236	31.5	37.3	24.6	20.8	26.3	19.9	8.1
Health and climate change: policy responses to protect public health	2015	481	146	30.4	28.1	31.5	14.4	25.3	27.4	19.9
Climate change impacts on bumblebees converge across continents	2015	337	101	30.0	34.7	27.7	16.8	12.9	19.8	14.9
Analysis and valuation of the health and climate change cobenefits of dietary change	2016	659	200	30.3	29.5	34.5	18.5	21.5	21.0	16.0
Oxygen isotope in archaeological bioapatites from India: Implications to climate change and decline of Bronze Age Harappan civilization	2016	537	114	21.2	47.4	12.3	18.4	12.3	25.4	8.8
Global and regional health effects of future food production under climate change: a modelling study	2016	347	100	28.8	37.0	23.0	12.0	23.0	26.0	18.0
Ecological networks are more sensitive to plant than to animal extinction under climate change	2016	276	59	21.4	18.6	59.3	13.6	10.2	18.6	13.6
Assessing the Performance of EU Nature Legislation in Protecting Target Bird Species in an Era of Climate Change	2016	238	77	32.4	32.5	27.3	7.8	11.7	33.8	18.2
